# Comparison of the effects of Ginger extract with clomiphene citrate on sex hormones in rats with polycystic ovarian syndrome

**Published:** 2017-09

**Authors:** Shekoufeh Atashpour, Hossein Kargar Jahromi, Zahra Kargar Jahromi, Mozhgan Maleknasab

**Affiliations:** 1 *Research Center for Non-Communicable Disease, Jahrom University of Medical Sciences, Jahrom, Iran.*; 2 *Department of Pharmacology, Jahrom University of Medical Sciences, Jahrom, Iran.*; 3 *Zoonoses Research Center, Jahrom University of Medical Sciences, Jahrom, Iran.*; 4 *Student Research Committee, Jahrom University of Medical Sciences, Jahrom, Iran.*

**Keywords:** Ginger, Clomiphene citrate, Polycystic ovarian syndrome

## Abstract

**Background:**

Polycystic ovarian syndrome (PCOS) is the most common endocrine disorder in women which affect fertility. Clomiphene citrate is used as first-line treatment for this disorder, which is associated with some complications and therapeutic resistance.

**Objective:**

In this research, we compare the effectiveness of ginger with clomiphene on sexual hormones such as Luteinizing hormone (LH), Follicle-stimulating hormone (FSH), estrogen and progesterone in order to treat PCOS effectively with fewer side effects.

**Materials and Methods:**

In this experimental study, 63 adult female rats (170-200 gr) were studied and divided randomly into 9 groups as control (not received any interventional substance for 60 and 89 days), sham (were given distilled water and ethyl alcohol intraperitoneally daily for 60 and 89 days), and 7 experimental groups receiving estradiol valerate (PCOS inducing agent, intramuscular) alone and with 100 mg/kg clomiphene or different doses of ginger extract (175 and 350 mg/kg) orally daily for 60 and 89 days. Sexual hormones were analyzed and compared in different groups.

**Results:**

Our results showed that in the PCOS-induced group, LH and estrogen concentration increased while progesterone and FSH concentration decreased remarkably (p<0.05) as compared to control group. Furthermore, in groups receiving clomiphene and ginger extract, we demonstrated significant (p<0.05) improvement in hormonal secretion as compared to the PCOS-induced group. Clomiphene, compared with the lower dose of ginger extract, had a better improving effect on balancing sexual hormones in PCOS. Moreover, ginger extract at higher doses has better effects in improving PCOS.

**Conclusion:**

As the long-term administration of clomiphene citrate has some side effects, the use of ginger as a herbal medicine without any side effects at high doses can be an effective and good alternative in improving PCOS.

## Introduction

Female reproductive system disorders such as hormonal and ovarian tissue disorders and how to prevent and treat them are one of the most important issues that researchers have now focused on (1, 2). One of the major causes of ovulation disorders is polycystic ovarian syndrome (PCOS) which is the most common endocrine disorder in women. Ovulation disorders account for about 30-40% of all infertility cases in women (3). Metabolic disorders including increased serum levels of Luteinizing hormone (LH), testosterone and prolactin is very common in women with PCOS and could influence women health in long-term (4-6).

One of the most common therapies is the administration of clomiphene citrate with an injection of human chorionic gonadotropin (7). Clomiphene can be used to improve ovarian function, menstrual pattern, and glucose metabolism in women with PCOS (2, 8). As clomiphene has structural similarity to estrogen compounds it could have negative effects on endometrial thickness (1, 8). On the other hand, long-term administration of chemical drugs could cause various side effects on human body and today experts believe that we should direct the patient to use herbal medications with lower side effects (1, 2).

Zingiber officinale Roscoe (family, Zingiberaceae), known as ginger, is consumed worldwide as a flavoring agent and medicine for thousands of years (9). In Ayurveda, ginger has been used as a carminative, sweat-inducing, anti-seizure, and blood circulation stimulator for the treatment of inflammation and rheumatoid arthritis (10). The main medicinal value of ginger is due to gingerol and shogaol which have potent antioxidant activity. In addition, it contains zingerone and some oily resin called gingerin (11-13). It has been shown that ginger could have a good effect in menstrual irregularities treatment and can inhibit ovarian cancer cells (14-16). Furthermore, some studies showed that ginger could enhance fertility index, serum testosterone level, testis and seminal vesicle weight, sperm motility, count, and quality and enhance male fertility in rats (17, 18). 

So in this study, we decided to compare the effects of different doses of clomiphene citrate and ginger extract in the treatment of PCOS in female rats.

## Materials and methods

**Chemicals**

Clomiphene was purchased from Sigma Company (Germany). LH, FSH, estrogen and progesterone measurement kits were purchased from Diameter Company (Italy).

**Collection and extraction of ginger**

Ginger rhizome was washed and dried in the laboratory and mixed with ethanol 70 (ethanol 70 and distilled water in 50:50 proportion), mixed for 24 hr at room temperature and a homogeneous mixture (Eidolph UNImax, 2010, Germany) was obtained. Then, the uniform solution was filtered and dried for 48 hr to obtain solid extract without ethanol. For drying and preparation of pure extract powder, the extract was placed in water bath and desiccator for 24 hr each in order to evaporate alcohol and water. The final dried extract was dissolved with distilled water. To prevent contamination, the extract stored in the refrigerator. Ginger doses were selected based on previous studies done on this herbal treatment. In this study, a minimum (175 mg) and maximum dose of ginger extract (350 mg) were selected which were extractedfrom previous study (19).

**Experimental animals**

63 adult female Wistar rats (weight: 170-200 gr, Age: 7-8 wk) were obtained from the Jahrom University of Medical Science’s animal house. The animal house temperature was maintained at 22±2^o^C with a 12 hr light/dark cycle. All animals were kept for 2 weeks prior to the experiment and had free access to food and water.

**Experimental design**

This is an experimental and randomized study. This study was performed in 2016 at Jahrom University of Medical Sciences. The rats were divided randomly into 9 groups (n=7/each) as followed:

Two control groups: rats did not receive any interventional substance during the study for 60 and 89 days, respectively.

Two sham groups: rats were given distilled water and ethyl alcohol (0.2 ml/ the ratio of 50: 50) intraperitoneally daily for 60 and 89 days, respectively.

Experimental group 1: rats were given estradiol valerate (used for induction of PCOS in female rats) once at the dose of 4 mg/kg intramuscularly and after 60 days the blood sample was collected.

Experimental group 2: rats were given estradiol valerate (single dose, 4 mg/kg) intramuscularly and after 89 days the blood sample was collected.

Experimental group 3: rats were given estradiol valerate (single dose, 4 mg/kg) intramuscularly and then clomiphene (100 mg/kg/day) orally daily for 88 days.

Experimental group 4: rats were given estradiol valerate (single dose, 4 mg/kg) intramuscularly and then ginger extract (175 mg/kg/day) orally daily for 88 days.

Experimental group 5: rats were given estradiol valerate (single dose, 4 mg/kg) intramuscularly and then ginger extract (350 mg/kg/day) orally daily for 88 days.

**Blood sampling**

The day after the end of the study (day 60 and 88) blood samples were collected from rats’ hearts directly using 5 cc syringes (rats were anesthetized by barbiturate), blood serum was collected after centrifugation (15 min, 3000 rpm) and stored at -20^o^C until they were tested. Biochemical measurement kits (Diametra, Italy) using the colorimetric method and an autoanalyzer machine (Selectera XL model made in Holland) were used for analyzing of sexual hormones including LH, FSH, estrogen and progesterone.

**Ethical consideration**

All procedures involving animals were reviewed and approved by the Institutional Animal Care and Use Committee (IACUC) of Jahrom University of Medical Sciences (IR.JUMS IR.JUMS.REC.1394.145).

**Statistical analysis**

All values were given as mean±SEM. Statistical analysis was carried out using Statistical Package for the Social Sciences (SPSS), version 21.0, SPSS Inc, Chicago, Illinois, USA, One-way analysis of variance (ANOVA) followed by Duncan post hoc test. Statistical p<0.05 was considered significant.

## Results

**Effect of different treatments on LH serum concentration**

**Figure 1:**
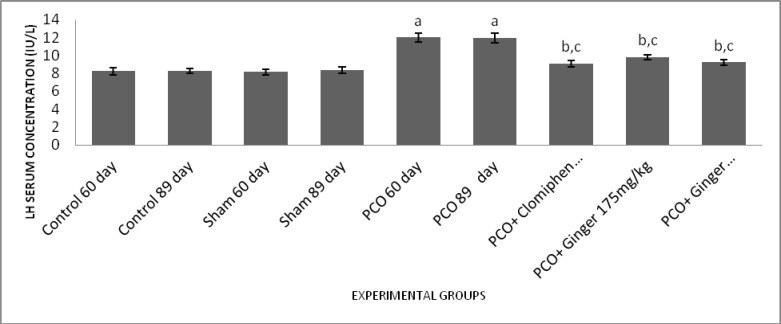
Effect of estradiol valerate, clomiphene and ginger extract on serum levels of LH. All data represent Mean±SEM. a: Significant difference (p<0.001) between PCO groups and sham/control groups, b: Significant difference (p<0.001) between treatment groups and sham/control groups, c: Significant difference (p<0.001) between PCO groups and treatment groups

The results of this study showed that LH serum level was significantly increased in the sham group (89 days) compared to control groups and sham group (60 days) (p<0.001). Also in experimental groups 1 and 2 (60 and 80 days) a significant increase in LH serum level was observed compared to control and sham groups (p<0.001). On the other hand, experimental groups 3, 4, and 5 showed a significant decrease in the level of LH compared to experimental group 1 and 2 (p<0.001) (Figure 1).

**Effect of different treatments on FSH serum concentration**

The results of serum concentration measurement of FSH showed that in experimental groups 1 and 2, FSH significantly reduced compared to control and sham groups (p<0.001). Furthermore, in experimental groups 3,4 and 5 showed a significant increase in FSH serum concentrations compared to experimental groups 1 and 2 (p<0.001). 

Moreover, in experimental group 5, FSH serum level significantly increased compared to experimental group 1 (p<0.001). These results showed that clomiphene and ginger extract could have positive and dose-dependent effect in PCOS treatment. In addition, the experimental group 5 showed a higher increase in FSH serum level compare to the experimental groups 4. 

**Effect of different treatments on estrogen serum concentration**

The results of our study showed that estrogen serum level increased significantly in all 5 experimental groups compared to control and sham groups (p<0.001). This showed that in PCOS, estrogen level will increase. On the other hand, in the experimental groups 3, 4 and 5 significant decrease in levels of estrogen were seen compared to Experimental groups 1 and 2 (p<0.001) (Figure 3).

**Effect of different treatments on progesterone serum concentration**

The results of our study showed that progesterone serum level decreased significantly in all 5 experimental groups compared to control and sham groups (p<0.001) which showed that PCOS can cause a reduction in progesterone serum level. 

On the other hand, in experimental groups 3, 4 and 5, significant augmentation in serum levels of progesterone was seen compared to experimental groups 1 and 2 (p<0.001) (Figure 4). Moreover, in experimental group 5 significantly higher increasing effect on progesterone serum level was seen compared to experimental group 4 (Figure 4).

**Table I:** Serum LH, FSH, estrogen, progesterone levels in the studied rats

**Groups**	**Hormones**			
	**LH (IU/L)**	**FSH (IU/L)**	**Estrogen (pg/ml)**	**Progesterone (ng/ml)**
Control (60 day)	8.22 ± 0.42	11.94 ± 0.41	264.04 ± 5.59	372.16 ± 4.47
Control (89 day)	8.28 ± 0.24	12.66 ± 0.39	259.88 ± 5.43	369.3 ± 7.91
Sham (60 day)	8.14 ± 0.34	11.38 ± 0.56	250.54 ± 5.82	366.76 ± 5.62
Sham (89 day)	8.36 ± 0.32	11.6 ± 0.51	262.64 ± 5.69	365.8 ± 5.56
Experimental group 1	12 ± 0.46 a	6.34 ± 1.15 a	372.64 ± 5.12 a	251.54 ± 6.77 a
Experimental group 2	11.94 ± 0.53 a	4.78 ± 0.23 a	375.34 ± 5.33 a	249.94 ± 2.99 a
Experimental group 3	9.06 ± 0.36 b, c	9.96 ± 0.28 b, c	281.58 ± 5.11 b, c	320.3 ± 4.16 b, c
Experimental group 4	9.8 ± 0.28 b, c	6.7 ± 0.43 b, c	322.48 ± 3.43 b, c	291.94 ± 7.94 b, c
Experimental group 5	9.22 ± 0.33 b, c	9.08 ± 0.66 b, c	299.58 ± 8.65 b, c	323.08 ± 3.79 b, c
P-value	<0.001	<0.001	<0.001	<0.001

All data are presented as the Mean ± SEM.

Statistical analysis was carried out using One-way analysis of variance (ANOVA) followed by Duncan post hoc test.

Statistical p<0.05 was considered significant

LH= Luteinizing hormone

FSH= Follicle stimulating hormone

a : Significant difference (p<0.001) between PCO groups and sham/control groups,

b : Significant difference (p<0.001) between treatment groups and sham/control groups,

c : Significant difference (p<0.001) between PCO groups and treatment groups.

**Figure 2:**
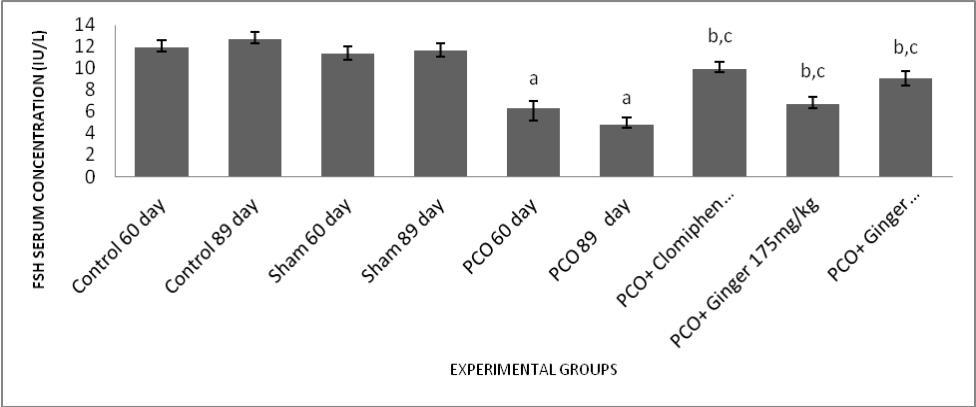
Effect of estradiol valerate, clomiphene and ginger extract on serum levels of FSH. All data represent Mean±SEM. a: Significant difference (p<0.001) between PCO groups and sham/control groups, b: Significant difference (p<0.001) between treatment groups and sham/control groups, c: Significant difference (p<0.001) between PCO groups and treatment groups

**Figure 3:**
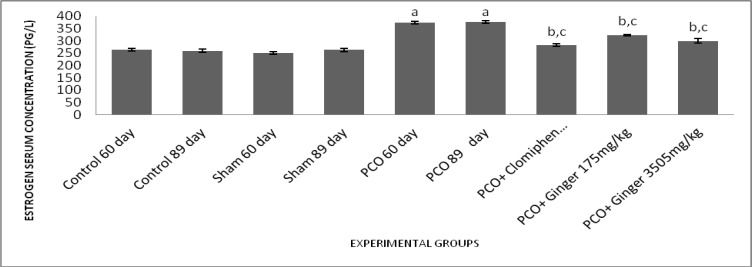
Effect of estradiol valerate, clomiphene and ginger extract on serum levels of estrogen. All data represent Mean±SEM. a: Significant difference (p<0.001) between PCO groups and sham/control groups, b: Significant difference (p<0.001) between treatment groups and sham/control groups, c: Significant difference (p<0.001) between PCO groups and treatment groups

**Figure 4:**
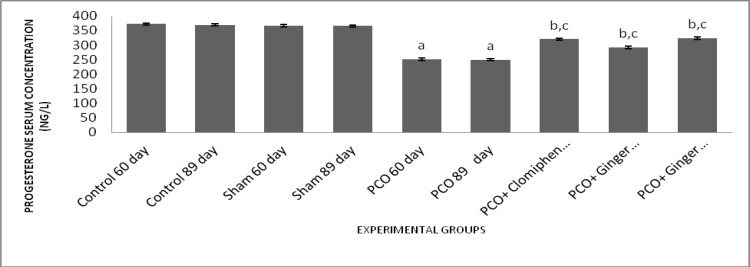
Effect of estradiol valerate, clomiphene and ginger extract on serum levels of progesterone. All data represent Mean±SEM. a: Significant difference (p<0.001) between PCO groups and sham/control groups, b: Significant difference (p<0.001) between treatment groups and sham/control groups, c: Significant difference (p<0.001) between PCO groups and treatment groups

## Discussion

The researchers found that one of the most important diagnostic criteria for the polycystic ovarian syndrome is changes in the level of sex hormones. In women with this syndrome, the serum level of testosterone, estradiol, and LH will increase and level of progesterone and FSH will decrease. In some cases, the serum level of FSH will not change in PCO disorder (20). Previous researches stated that hyperandrogenism and increased serum levels of LH are very common in PCOS (21). Some other reviews demonstrated that PCOS could increase secretion of testosterone and LH and reduce FSH hormones secretion (22). One possible mechanism for an explanation of sex hormones changes in PCOS is relative lack of aromatase enzyme in the ovary which could increase androgen concentration (23).

Furthermore, androgens could enhance the level of FSH receptors in women with PCOS and thereby cause a reduction in the concentration of this hormone and augmentation of LH serum level (23). The present study demonstrated a significant increase in LH and estrogen serum level and remarkable reduction in FSH and progesterone serum level in PCO-induced groups compared to control group which indicates the negative effect of this disorder on estrogen, progesterone and gonadotropin hormones. These changes were in accordance with those previous studies and probably these changes in hormonal level in PCOS are due to a mutation in aromatase enzyme (23).

Previous studies on LH and FSH serum level changes in PCOS illustrated that there is a connection between the increased level of LH/ reduced level of FSH in PCOS and insulin resistance. Enhanced level of LH hormone stimulates ovarian theca cells, which in return stimulates androgen production (24, 25). In the present study, we also reported enhancement in serum level of LH and estrogen hormone and reduction in the level of FSH and progesterone which were in concurrence with previous studies. On the other hand, any agent that could reduce estrogen and LH and increase FSH and progesterone level can be used to treat this disorder. 

The results of this study showed that clomiphene citrate could significantly decrease estrogen and LH serum level and remarkably increase progesterone and FSH serum level compared to PCO-induced groups (with no treatment) which indicate the useful effect of clomiphene citrate in improving hormonal changes in rats with PCOS. These results were supported by previous studies (26, 27). 

Clomiphene citrate has some structural similarity to estrogen compounds and by occupying estrogen receptors could decrease the function of estrogen and increase progesterone performance (27). Furthermore, clomiphene by having an anti-estrogenic effect could stimulate the pituitary Gonadotropin-releasing hormone secretion, regulate gonadotropin secretion and ultimately stimulate ovulation (1). Therefore, it is likely that clomiphene could improve the sex hormone changes in women with PCOS due to having anti-estrogenic properties, which is consistent with our results. Other studies had suggested that clomiphene citrate will improve ovulation and fertility in women with PCOS (28, 29). 

Also, previous studies suggested that clomiphene citrate is more effective than similar drugs in the treatment of PCOS (30). In the current study, improvement in sex hormone serum level was higher in rats receiving clomiphene citrate than the ginger-exposed group, which indicates that clomiphene is more effective in improving sex hormone changes in patients with PCOS. Moreover, our study showed that ginger extract could improve sex hormone changes compared to the PCO-induced group with no treatment. We observed that ginger extract could have positive and dose-dependent effect in improving serum level changes of LH, FSH, estrogen and progesterone in PCOS. Although ginger extract is more effective in improving hormonal changes at higher doses, even at the highest dose, it has less improving effect compared to clomiphene. 

In addition, results of the current study showed that serum concentration of LH and estrogen hormone significantly decrease in the group receiving ginger extract compared to non-treated PCO group while ginger could remarkably increase serum level of FSH and Progesterone. These results confirm the positive effect of ginger extract in the treatment of PCOS. Previous studies suggested that ginger extract contains active ingredients such as gingerols and sesquiterpenes which inhibit arachidonic acid production by interfering with lipoxygenase and cyclooxygenase pathways and thereby inhibit prostaglandin production (12, 31). 

In turn, this effect regulates gonadotropin production (12). So it is likely that changes in gonadotropin level in this study be due to possible effects of ginger extract active ingredient on prostaglandins, which is consistent with results of previous research. So we can suggest ginger extract as adjunctive therapy for improvement of gonadotropin hormones level in patients with PCOS. On the other hand, it has been stated that in patients with PCOS, the levels of progesterone decreased while estrogen levels increased (32).

Thus, any agent that could regulate the secretion of these hormones can be effective in the treatment of PCOS. Studies had shown that ginger extract also contains many flavonoids and polyphenolic compounds which previously mentioned (31). Researches had suggested that polyphenols have anti-androgenic effects. Evaluation of other plants with similar compositions to ginger extract demonstrated that these herbal extracts could reduce estrogen level compared to PCOS group and this effect is mostly due to flavonoids and phytoestrogens component of the extract which reduces aromatase enzyme activity and thus reduce estradiol concentration. On the other hand, it has been expressed that plants with similar compositions with ginger extract will reduce cholesterol level and as steroid hormones such as estradiol are derived from cholesterol, this reduction in cholesterol concentration could decrease synthesis of steroid hormones such as estradiol which was in accordance with estrogen serum level changes by ginger extract in the current study (33). 

It is also indicated that in PCOS rats some changes in estrogen and progesterone level will occur as progesterone to estrogen ratio will reduce and balance of these hormones can be one way of treating this disorder (33). Results of the current study showed that serum level of progesterone significantly increased in ginger-treated group compared to PCOS group which indicates the useful effect of ginger in PCOS treatment.

Studies on plants with similar compositions to ginger extract exerted that plants with flavonoids and phenolic compounds could establish a natural balance between estrogen and progesterone and with their specific pharmacological-physiological effects could rebalance increase or decrease of sex hormone (34, 35). So it is possible that ginger extract could make a balance between estrogen and progesterone hormones in PCO-induced rats via having flavonoid content and antioxidant activity which is inconsistent with the results of previous studies. So dose-dependent increase in progesterone and decline in estrogen level seen in the group receiving ginger extract in comparison to PCOS groups can represent balancing properties of ginger extract in this disorder.

## Conclusion

The results of the current study showed that clomiphene citrate, compared with ginger extract, has more improving and effective properties in balancing LH, FSH, estrogen and progesterone hormones in rats with polycystic ovarian syndrome. ginger extract at higher doses also has a better effect in improving this disorder so that ginger extract in high doses has an almost similar function with clomiphene citrate. As the long-term administration of clomiphene citrate has some side effects, the use of ginger as a herbal medicine without any side effects at high doses can be an effective and good alternative in improving PCOS.

## Note

***This article extracted from M.D. thesis. (Mahnaz Maleknasab)***
